# Recent advances in urodynamics in women

**DOI:** 10.12688/f1000research.24640.1

**Published:** 2020-06-15

**Authors:** Georgina Baines, Ana Sofia Da Silva, George Araklitis, Dudley Robinson, Linda Cardozo

**Affiliations:** 1 Department of Urogynaecology, King's College Hospital, London, UK

**Keywords:** Urodynamics, Advances

## Abstract

Urodynamics is the study of the storage and evacuation of urine from the urinary tract. The aim is to reproduce the patient’s symptoms and provide a pathophysiological explanation for them by identifying all factors that contribute to the lower urinary tract dysfunction, including those that are asymptomatic. Urodynamics consists of various tests, each of which is designed to assess a different aspect of lower urinary tract function. There is a lack of evidence regarding when urodynamics should be used in the non-neurogenic bladder. Some small randomised controlled trials suggest that urodynamics does not alter the outcome of surgery for stress urinary incontinence when compared with office evaluation alone. However, this is widely felt to be inaccurate and many health-care professionals still advocate the use of urodynamics prior to any invasive treatment, especially surgery on the lower urinary tract. There have been few technological advances in urodynamics in recent years. Air-charged rather than fluid-filled catheters were thought to help reduce artefact, but the evidence is unclear, and there is doubt over their accuracy. Ambulatory urodynamics is carried out over a longer period of time, enabling physiological bladder filling, but it remains invasive and artificial. To attempt to replicate symptoms more accurately, there have been efforts to develop wireless devices to measure detrusor pressure directly. These may be promising but are far from suitable in humans at present. Urodynamics continues to provide useful information for assessing lower urinary tract function, but further large studies are required to assess its value and develop innovations to improve the accuracy of the tests and acceptability to patients.

## Introduction

Urodynamics (UDS) is a range of diagnostic tests that are used widely by urologists and gynaecologists to assess the function of the lower urinary tract.

### History

UDS was first described in the 1800s when interest in the relationship between bladder pressure and urine flow began to develop. The term ‘urodynamics’ was first used by Davis in 1953 to describe the function of the urinary tract ^1^. Over time, it was felt that an objective, scientific method of quantifying urinary tract function was essential and Hodgkinson stated that ‘to ignore this would be like treating a myocardial infarction without an ECG’ ^2^. In 1970, Bates *et al*. described the bladder as ‘an unreliable witness’ ^3^ and this was supported by work in the ’80s which showed a poor correlation between the symptoms reported by patients and their urodynamic diagnoses ^4– 6^.

### Aim of urodynamics

UDS is a collective term to describe investigations to assess the ability of the bladder and urethra to store and expel urine ^6^. The aim is to reproduce the patient’s symptoms and provide a pathophysiological explanation for them by identifying all factors that contribute to lower urinary tract dysfunction (LUTD), including those that are asymptomatic.

When a patient presents with LUTD, she should first have a thorough history taken to include all co-morbidities and medications. It is important to ascertain the level of function of the patient, the impact that the LUTD has on her quality of life and her expectations of treatment. An important component of this is the bladder diary ^7^. For this, the patient is required to record fluid intake and output, urgency, and incontinence episodes for three days. This can give the clinician an idea of how to direct further investigations and what conservative management may have the best effect. Alongside the bladder diary, patient-reported outcomes can be standardised by using validated questionnaires. The International Consultation on Incontinence have developed 16 questionnaires that when carefully selected can help characterise LUTD and also provide tools for standardisation in reporting clinical trials ^7^.

Every patient should undergo urine dipstick testing to rule out urinary tract infection and identify any microscopic haematuria that may require further investigation.

Physical examination is essential in ensuring that there are no obvious anatomical abnormalities to explain LUTD; however, there are no studies that support evidence that examination alone can help in the classification of LUTD ^7^. Particular features to note on examination include any pelvic organ prolapse, vulvo-vaginal atrophy or excoriation and scars from previous surgery.

UDS may be used following these initial steps to help clarify a diagnosis, predict the consequences of treatments for LUTD and evaluate when therapies have failed. It also provides an objective and reproducible measurement for assessing the impact of novel or experimental treatments ^7^.

## What is urodynamics?

The term ‘urodynamics’ encompasses a range of investigations that aim to characterise lower urinary tract function. Some or all of the following components may be used to diagnose LUTD.

### Uroflowometry

Uroflowometry refers to the non-invasive measurement of urine flow rate. This is undertaken by asking the patient to void onto a flow meter which generates a graph of volume voided against time. From this, volume voided, peak flow rate and flow pattern can be determined. Women normally void at a maximum flow rate greater than 15 mL/sec for a volume voided greater than 150 mL, although this decreases with increasing age. A low flow rate or an intermittent flow may represent voiding dysfunction which can be due to an underactive detrusor or outflow obstruction but the two cannot be differentiated by measurement of a flow rate alone.

### Filling cystometry

This part of UDS is an invasive test to determine the pressure generated by the detrusor muscle. A pressure transducer is inserted into the bladder (intra-vesical pressure) and another one is inserted into the rectum or vagina to measure intra-abdominal pressure. The detrusor pressure is calculated by subtracting the intra-abdominal pressure from the intra-vesical pressure which is displayed graphically.

The bladder is filled through a separate filling channel or catheter with saline or radio-opaque dye (for videourodynamics). This filling takes around 10 minutes and is therefore faster than would occur physiologically. Throughout filling, the detrusor pressure is monitored, and the patient is asked when she feels the first sensation (normally 150–250 mL) of bladder filling and a strong desire to void. The cystometric capacity can be determined as the point when the patient cannot tolerate further filling (normally 400–600 mL). During filling, sensation, compliance and spontaneous or provoked detrusor contractions are recorded.

Following filling, coughs can be used to determine whether urodynamic stress incontinence (USI) is present (leakage of urine in the absence of a detrusor contraction), and various techniques such as placing the patient’s hands in cold water to provoke uninhibited detrusor contractions can also be used.

Throughout filling cystometry, detrusor pressure should remain low (less than 15 cm H _2_O to a volume of 500 mL). Any increase in the detrusor pressure above this, representing spontaneous, uninhibited detrusor contractions, are considered pathological and leads to a diagnosis of detrusor overactivity (DO).

### Voiding cystometry

This gives an insight into the mechanics of micturition. The patient is given permission to void onto a flow meter with the pressure catheters *in situ* so the relationship between pressure and flow can be calculated. This study is particularly useful in diagnosing the causes of voiding dysfunction, such as urethral stricture (high detrusor pressure and low flow rate) or an underactive detrusor (low detrusor pressure, low flow rate, and increase in abdominal pressure used to void). An important aspect of voiding cystometry is measurement of the urinary residual immediately post-void by in-out catheterisation (normally less than 100 mL).

### Urethral pressure profilometry

This test measures the pressure in the urethra relative to the bladder. It is assumed that the increase in urethral pressure is generated by the resting tone of the urethral sphincter. It is demonstrated by slowly withdrawing a pressure transducer through the urethra and observing the changes in pressure along its length. The maximum pressure elicited is presumed to be the closing pressure of the urethra. A low value should not be used to diagnose stress urinary incontinence (SUI) since it correlates poorly with severity of symptoms ^8^.

We use two transducers 6 cm apart in order to subtract bladder pressure from urethral pressure. Urethral pressure profilometry can also be carried out by using water-perfused catheters.

### Videourodynamics

This is a specialised version of cystometry by combining it with visualisation of the lower urinary tract. The bladder is filled with radio-opaque dye instead of saline, which enables bladder morphology to be determined during the test by radiological imaging. This is particularly useful in the diagnosis of diverticula, fistulae, and ureteric reflux and in patients with neurological and complex disorders.

### Ambulatory urodynamics

This test is based on the same principles as traditional UDS but relies on physiological bladder filling. It takes much longer for the patient to reach cystometric capacity and allows time for the patient to ambulate and recreate situations when she suffers from incontinence. This may detect abnormal findings (especially DO) more than standard UDS, but the clinical significance is unclear ^9, 10^.

A working group was set up in 2002 by the International Continence Society (ICS) to set standards for UDS equipment and technique for the purpose of ensuring that tests are accurate and reproducible across different units. The most recent document was published in 2017 ^11^ (see also 12, 13).

## Indication for urodynamics

UDS has traditionally been used to obtain an objective diagnosis for a subjective condition with the aim of tailoring treatment most effectively. There are a number of guidelines pertaining to the indication for UDS:

The ICS advises UDS in patients with symptoms (particularly incontinence) that have both stress and urgency characteristics or include nocturnal enuresis. It is also indicated in patients who present with persisting symptoms despite initial management or who express the wish for more invasive/irreversible treatment ^7^.

The European Association of Urology guidelines recommend UDS if the findings may influence the choice of invasive treatment; it also recommends that UDS not be routinely offered for uncomplicated incontinence or prior to treatment of pure SUI.

National Institute for Health and Care Excellence (NICE) guidelines advise that UDS should not be undertaken prior to primary SUI surgery if the main symptom is SUI or stress-predominant mixed incontinence. However, it is advised that they be performed prior to surgery if urgency is the predominant symptom or there is any voiding dysfunction, previous surgery for SUI or apical/anterior compartment vaginal prolapse ^14^.

It is clear that UDS should be used when the diagnosis is unclear or invasive treatments are planned; however, the value of UDS in women presenting with pure SUI has been debated in the literature and is contentious.

A study of women undergoing UDS showed that 78% of women presenting with a history of pure SUI had this confirmed on UDS ^15^. The authors felt that, in the absence of urodynamic investigation, 22% of these women would be over-treated ^15^. These data were supported by an Italian study of women undergoing UDS prior to SUI surgery ^16^. The results of the UDS changed the management in 19% of cases because of re-classification of the type of incontinence ^16^. Another Italian study performed UDS in a group of women with pure SUI ^17^. In that group, 74% demonstrated pure USI and went on to have surgery; 91.6% reported their symptoms as ‘cured’. The rest of the patients who demonstrated pure DO, mixed DO and USI or inconclusive findings were managed with anti-muscarinics, and the cure rate was 49.2%; 19% went on to have surgery for SUI ^17^. This trial implies that a UDS diagnosis of USI is a good way of predicting which patients will benefit from more invasive treatment in the form of surgery and those who will be managed well with medication.

Two important trials helped shape the guidelines regarding pre-operative UDS in pure SUI. The first was the Value of Urodynamic Evaluation (ValUE) trial. In this trial, 630 women with a history of ‘uncomplicated SUI’ were randomly assigned to pre-operative UDS or surgery alone following office evaluation. The investigators found that pre-operative UDS increased the clinician’s confidence in their diagnosis but did not alter the treatment success (patient-reported outcomes). Interestingly, women undergoing UDS were less likely to receive a diagnosis of overactive bladder and more likely to receive a diagnosis of voiding phase dysfunction. The authors concluded that office evaluation alone was non-inferior to UDS in the pre-operative assessment of SUI ^18^.

The Dutch study is called the Value of Urodynamics prior to Stress Incontinence Surgery (VUSIS I and II) were multi-centre, non-inferiority trial. The second consisted of two studies carried out in the Netherlands; VUSIS I and II. These were both multi-centre, non-inferiority trials ^19^. They confirmed the findings that empirical treatment of pure SUI is non-inferior to pre-operative UDS ^19^.

A 2015 meta-analysis identified only four randomised controlled trials (RCTs) and concluded that pre-operative UDS in women with pure SUI or stress-predominant mixed incontinence did not improve objective or subjective outcomes ^20^.

It is unclear how this evidence has shaped clinical practice. A survey of Dutch gynaecologists and urologists revealed that only 7% of clinicians now perform UDS in this group of patients whereas 37% did so previously ^21^.

There is growing concern that this decrease in use of UDS is unjustified. Critics of this change in practice feel that the RCTs were flawed. The studies were too small (in particular, VUSIS, where n = 109) and included only women with SUI when it is known that the proportion of women presenting with this is low. There are concerns that UDS were not standardised across sites and their interpretation was unclear and subjective. Voiding dysfunction was shown to be a significant diagnosis in the UDS group, which otherwise may be undiagnosed and could present with complications in the future ^22^.

The guidelines described apply to the non-neurogenic bladder, and it should be noted that in patients who have an underlying neurological diagnosis, UDS is essential to help guide treatment and advise on prognosis.

Whilst there is ongoing debate regarding the clinical value of UDS, the mechanics of the test have had few innovations in recent years.

The ICS used an evidence-based strategy to develop a guide for good urodynamic practices ^11^. These are regularly updated to incorporate any modernisation in technique or equipment. A challenge in the practice of UDS is to maintain the accuracy and fidelity of the test by ensuring that pressure changes can be recorded and interpreted correctly and attempts have been made to improve this.

## Types of pressure transducers

The ICS has defined the standard catheter used during UDS as a water-filled catheter with an external pressure transducer. In the 1970s, air-charged catheters (ACCs) were suggested as a way of reducing artefact due to patient movement ^23^.

A review of the available evidence for air-filled catheters found that there was a significant difference between pressures recorded by the different types of catheters and that the cause for this is unclear ^24^. One laboratory-based study suggested that bladder pressure changes were reported in very different ways by the different types of catheter ^25^. Water-filled systems are vulnerable to excessive artefact, but ACCs can ‘over-attenuate’ artefact and cancel signals that are high-frequency ^25^. Only two *in vivo* studies were identified. One reported that differences of up to 10 cm H _2_O were found between the different systems and advised that if ACCs are to be used, normal resting pressures will have to be re-established ^26^. Another clinical study confirmed that ACCs gave readings that were significantly different from those of water-based systems ^27^.

There is not sufficient evidence to recommend the use of ACCs for the measurement of pressures during UDS in women, but ACCs may be useful in reducing artefacts which make traces more difficult to interpret. Further work is required to establish the accuracy of this technology and its application in clinical practice.

An alternative pressure sensor is one that sits on the tip of the catheter. The advantage of this system is that no water flushing of the tubes is required, the patient is connected by wires rather than water and movement artefact is reduced. As opposed to water-filled systems that are cheap and disposable, these catheters are relatively fragile and require cleaning after each patient. There are few studies comparing water-based and catheter tip–mounted transducers but those that exist show that there may be poor correlation between readings from different transducer types but that reproducibility with an individual system is high ^28, 29^.

Where possible, water-filled catheters should be used for transducing intra-vesical and -abdominal pressures in line with ICS guidance. Other measuring systems may have advantages in eliminating movement artefact and the need for flushing (which may be uncomfortable to the patient). If alternative systems are used, repeated measurements should be made on the same equipment to ensure reproducibility of the results.

## Technological advances

In other areas of medicine and technology, devices are getting smaller, battery-operated, and less invasive. This may prove particularly useful in avoiding catheterisation in UDS since around 50% of patients undergoing UDS for the first time find catheterisation physically or emotionally uncomfortable ^30^.

There are efforts to develop less invasive methods of UDS with the aim of making a quicker or cheaper test or one that more closely reflects normal life ^31^.

Hydration studies looking at changing patterns of bladder sensation may provide insights into overactive bladder symptoms ^32^. Some more novel and less invasive methods of assessing bladder function include shear wave elastography ^33^, acoustic radiation force impulse imaging ^34^, ultrasound vibrometry, ^35^ and ultrasound bladder shape analysis ^36^.

A number of wireless devices (none approved for clinical use) that do not involve continuous catheterisation have been reported. These are implanted using the urethral or supra-pubic route into the bladder, into the detrusor, or across the detrusor ^37^. They enable the subject to ambulate and achieve physiological bladder filling without the artificial sensation of a catheter. Bioabsorbable technology can be incorporated to avoid the need for removal ^38^.

Whilst in theory this is promising, the invasive nature of the insertion of the devices may prevent them from being adopted in humans. Further research is needed to optimise minimally invasive devices, focussing on accuracy of measurements and acceptability to patients ^37^.

A recent study looked at using 2% lidocaine gel compared with a water-based lubricant for catheterisation showed a significant reduction in pain score during the procedure ^39^. Whilst the use of lidocaine gel may make catheterisation less painful, the study did not look at any effects on urodynamic parameters. Another study showed that 4% lidocaine instilled urethrally decreased flow rate (but not pain scores), implying a sensory role of the urethra in voiding ^40^. Therefore, we would caution against the use of local anaesthetic gel at the time of UDS unless necessary.

Another problem in ambulatory UDS is calculation of bladder volume. In conventional UDS, bladder volume can be estimated fairly accurately by the instilled volume because additional physiological diuresis will be small over a short space of time. In ambulatory UDS, the test relies on physiological filling. Ultrasonography (USS) has been shown to be an effective and non-invasive way of calculating bladder volume, and modern devices are becoming smaller and more mobile ^41^. The main disadvantage of this technique is that the devices require a high power consumption and therefore a non-mobile power source ^37^.

Near-infrared spectroscopy and bioimpedance are both non-invasive ways of estimating the change in bladder volume. They rely on the fact that the optical (infra-red spectroscopy) and electrical conductance (bioimpedance) properties of the pelvis are constant and that any changes are due to filling or emptying of the bladder ^42, 43^. Whilst both of these techniques are non-invasive and mobile, both have a low specificity and so may not be clinically useful at present ^37^.

## Research in urodynamics

In 2018, a think tank at the International Consultation on Incontinence Research Society acknowledged that there was a need for standardisation in research into UDS. This was following the growing concern of a lack of regulation in the introduction of surgical and diagnostic techniques. This is most evident when compared with the introduction of new pharmaceuticals which undergo rigorous testing before becoming widely available ^44^. The ‘IDEAL’ model was proposed to standardise the introduction of new surgical techniques. It involves a series of steps: innovation, development, exploration, assessment, and long-term study. The think tank reported that this approach should be adopted for UDS, meaning that any innovations should be tested in the laboratory before clinical trials or use in patients ^45^.

## Further assessment of the lower urinary tract

We should be mindful that UDS assesses only the function of the lower urinary tract. It does not replace other imaging modalities. Direct visualisation of the urinary tract with rigid or flexible cystoscopy is essential if any lower urinary tract malignancy is suspected. Cystoscopy can add to information gained from videourodynamics regarding anatomical abnormalities such as fistulae or diverticula.

Because of its low cost and safety to patients, USS is considered the ‘gold standard’ for imaging of the upper urinary tract. If USS is non-diagnostic, computed tomography (CT) or magnetic resonance imaging can be used to further delineate the urinary tract; however, the nephrotoxicity of contrast and electromagnetic radiation (for CT) should be considered before employing these modalities.

## Conclusions

UDS represents a range of investigations widely used across urology and gynaecology. It has an important role in managing patients with complicated urinary incontinence, failed previous surgery or an underlying neurological diagnosis. Its use prior to SUI surgery is unclear. A small number of RCTs support the fact that office evaluation alone is non-inferior to UDS, but there is a professional consensus that this may not be true. This area is in need of well-designed RCTs to give us more robust evidence to ensure that patients receive all necessary investigations without excessive intervention.

Invasive UDS should be carefully considered regarding benefits versus risks before being undertaken. Future work should look into non-invasive methods of measuring changes in bladder pressure and lower urinary tract function, emphasising the need for accuracy and reproducibility alongside being acceptable to the patient.
